# Influence of contextual variables and the pressure to keep category on physical match performance in soccer players

**DOI:** 10.1371/journal.pone.0204256

**Published:** 2018-09-20

**Authors:** Jorge García-Unanue, Jorge Pérez-Gómez, Jesús-Vicente Giménez, José Luis Felipe, Santiago Gómez-Pomares, Leonor Gallardo, Javier Sánchez-Sánchez

**Affiliations:** 1 Faculty of Sport Sciences, Universidad Europea de Madrid, Villaviciosa de Odón, Madrid, Spain; 2 Faculty of Sports Sciences, University of Extremadura, Cáceres, Spain; 3 Faculty of Physical Activity and Sport Sciences, Technical University of Madrid, Madrid, Spain; 4 IGOID Research Group, Universidad de Castilla-La Mancha, Toledo, Spain; Berner Fachhochschule, SWITZERLAND

## Abstract

Previous studies have analysed the influence of contextual variables on performance and physical demands in soccer. However, the points needed to remain in the category have been an element that has not been analysed previously. The aim of the present study was to investigate the influence of match location, match period, strength of the opponent and the points required to keep category on physical performance in professional soccer players. Fourteen Spanish second B Division League matches played by a professional football team were analysed during the 2016/17 season using GPS devices. The 10 main players of each match used the GPS throughout the match. The variables of Total Distance (m), High Intensity Distance (m), High intensity Accelerations (n), Sprint Time (s) and Sprint Distance (m) were analysed. The most notable differences are found in Total Distance covered. Away games accumulated significantly more distance than those played at home, but only in the second half (+230.65 m, IC95%: 21.94 to 438.19, ES: 0.46, *p* = 0.031). There are no differences depending on the strength of the opponent. However, players covered greater distances during the first half in those matches that were played furthest from salvation (+235.86 m, 95% CI: 49.03 to 422.70, ES: 0.51, *p* = 0.014). Total Distance is the main parameter affected by situational variables. In addition, the pressure of being further away from saving the category increases the distance covered by players in a game.

## Introduction

Recent advancements has shown the constraints to achieving sporting success, thus performance match analysis utmost importance [1,2]. For example, current literature supports that to achieve victory, players must possess a high level of physical and psychological conditioning, as well as appropriate technical-tactical preparation [3]. Therefore, it has been shown that matches are influenced by the specific technical, tactical and physical demands of soccer with aerobic training being a very important component for the physical condition of soccer players [4]. Thus, previously, researchers have proven the relevance of aerobic training, aerobic power, competitive ranking, the quality of the game and distance covered during a match as relevant factors on player’s performance [5].

In line with the above, it is known that GPS has become the indispensable tool to quantify the player's load for coaches and sport sciences. Thus, GPS, in professional soccer teams is a tool used to monitor the game, providing a reliable and valid measurement to control the load individually of the player and/or the group [6,7]. The fact that the technical staff can quantify the load of the training sessions and matches, adapting it when needed, leads to a minimization of injury risk, avoiding an inadequate distribution of the loads across the season [8].

The use of GPS systems demonstrates an increase in knowledge about the dynamics of the load, and in turn, this reveals the physical profile of the player and their evolution within different training and competitive contexts [9]. The possibilities of its use and the ability to study the dynamics of the load during matches, provides coaches with very relevant information of the physical demands in each scenario of the game, allowing for decisions to be made and avoiding guesses to be taken. Despite this, previous research has shown the importance of taking into account the different situational variables during matches, since these can influence the performance of the players [10–13]. In addition, it should be noted that performance indicators are defined as the selection and combination of variables that define some aspect of performance and help achieve athletic success [14,15]. Therefore, since soccer is dominated by strategic factors, it is reasonable to suggest that situational variables can in some way influence the performance of teams and their players [12]. Even so, previous research revealed that both the physical and technical-tactical actions of soccer are influenced by situational variables influencing players at a behavioral level [16], and association between outcomes (notational analysis) and processes (spatiotemporal analyses) may also contribute to identify which patterns can be avoided or reinforced to increase possibilities for success [15].

In this sense, some studies already follow that the variables of match location (playing home or away [12]), match period (first half or second half), the strength of the team and the opponent (high-level teams, intermediate-level teams or low-level teams [17]) all influence in the performance of the players. In addition, some studies suggest that professional soccer players, at an individual level, have the ability to regulate their physical effort in accordance with the specific demands required by the matches during different periods of the game. [18].

However, to our knowledge no studies have analyzed the effect of the amount of points needed to stay in the category or enter in relegation positions on physical performance in professional soccer players. One study has been conducted to analyze the effect of the teams ranking on ball possession in elite soccer players [19], although this is a tactical skill and not a physical performance. Therefore, there is still the need to clarify if the points required to keep category can influence the physical performance in professional soccer players.

Therefore, it is notable that the available research has not addressed the evaluation and performance of the teams according to the situational variables, acknowledging Performance Analysis (PA) as a social process given its integral role in the coaching process [20], and the pressure to keep category in professional soccer players with the ability to cope competitive stress and with imperative of winning to avoid relegation.Tthis aspect may be especially relevant since until now, it seems reasonable to hypothesize that the dynamics of the game is influenced by situational variables, despite the social and cultural influences that affect al PA delivery and athlete learning in applied setting [21].

The aim of the present study was to investigate the influence of the location of the game, the match period, the level of the opponent and the points necessary to keep category in relation to the physical variables in professional soccer players.

## Material and methods

### Subjects

Fourteen Spanish second B Division League matches played by a professional football team were analyzed during the 2016/17 season using a GPS system with 15 Hz sampling frequencies (GPSport, Australia). Second B Division League is the third national division, preceded by the first and second division. It is divided by four groups of twenty teams each one and include both professional and semiprofessional teams. The matches analyzed correspond to the analysis of observations of the 10 main players of each match of the same team (the goalkeeper was excluded) from match 22 to 35 of the 2016/2017 season. The Clinical Research Ethical Committee of Castilla-La Mancha Health Service (Spain) approved this study basing on the last version of the Helsinki Declaration.

### Design

The 10 main players of each match used the GPS throughout the match. All variables were calculated in two 45-minute splits, one for each part of the match. The sampled match results (7 home and 7 away matches) consisted of 2 wins, 5 draws and 7 loses, with a total of 11 goals scored and 18 conceded by the sample team. The teams overall statistics for the sample season were 5 wins, 13 draws, 20 loses, 36 goals scored, and 58 conceded. Only 14 out of the total of 38 matches of the season were analysed, choosing the key matches where the objective of the team was to reach the safety position to stay in the league. For this reason, the matches prior to the 22nd match and after match 35th (where fulfilling the aim of staying up was mathematically impossible) were ignored.

### Methodology

The GPS software (TeamAMS, GPSport, Canberra, Australia) provided information about the total distance covered during the game and the distance covered in each 1 of the 6 locomotor categories with speed ranges adapted from previous studies [22,23]: standing (0–2 km·h^-1^), walking (2–7 km·h^-1^), easy running (7–13 km·h^-1^), fast running (13–18 km·h^-1^), high-speed running (18–21 km·h^-1^), and sprinting (21 km·h^-1^). Actions above 18 km·h^-1^ were taken into account for this study (high-speed running, and sprinting). These variables were grouped and defined as high intensity running. The GPS software also provided information about the number and average distance of the sprints. Sprint time (s), is the average time that athletes are above 21 km·h^-1^ and sprint distance (m), is the distance covered with speed above 21 km·h^-1^. In the same way, the GPS devices registered the maximum acceleration peaks and the number of accelerations of the players in different ranges of intensity used in previous research [24]: 1.5–2, 2–2.5, 2.5–2.75, and 2.75 m·s^-2^. The variable High intensity accelerations are the accelerations made in the maximum intensity zone.

The rival teams were divided into three groups using the k-mean cluster analysis to identify the opponents quality (using their total points at the end of the season as the reference variable) and also two groups taking into account their proximity to saving the category (using as a reference the remaining points needed to reach the position in the classification necessary to keep category before each game). These data were analyzed for home and away matches.

### Data analysis

Results are presented as means (M) ± standard deviations (SD). Normality and homogeneity of the variance were assumed after the Kolmogorov Smirnov test and the Levene’s statistical analysis. Three comparison analysis between performance variables were developed through two-way ANOVA tests and Bonferroni post-hoc (home vs away matches and first vs second half; closeness to remaining in the category and first vs second half; quality of the opponent and first vs second half). Confidence interval (CI of 95%) was included to identify the magnitude of changes. Effect sizes (ES) were calculated and defined as follows: trivial, <0.19; small, 0.2–0.49; medium, 0.5–0.79; large, >0.8 [25]. Data were analyzed with the statistical software SPSS v20.0. The level of significance was established at *p*<0.05.

## Results

**Fig 1:**
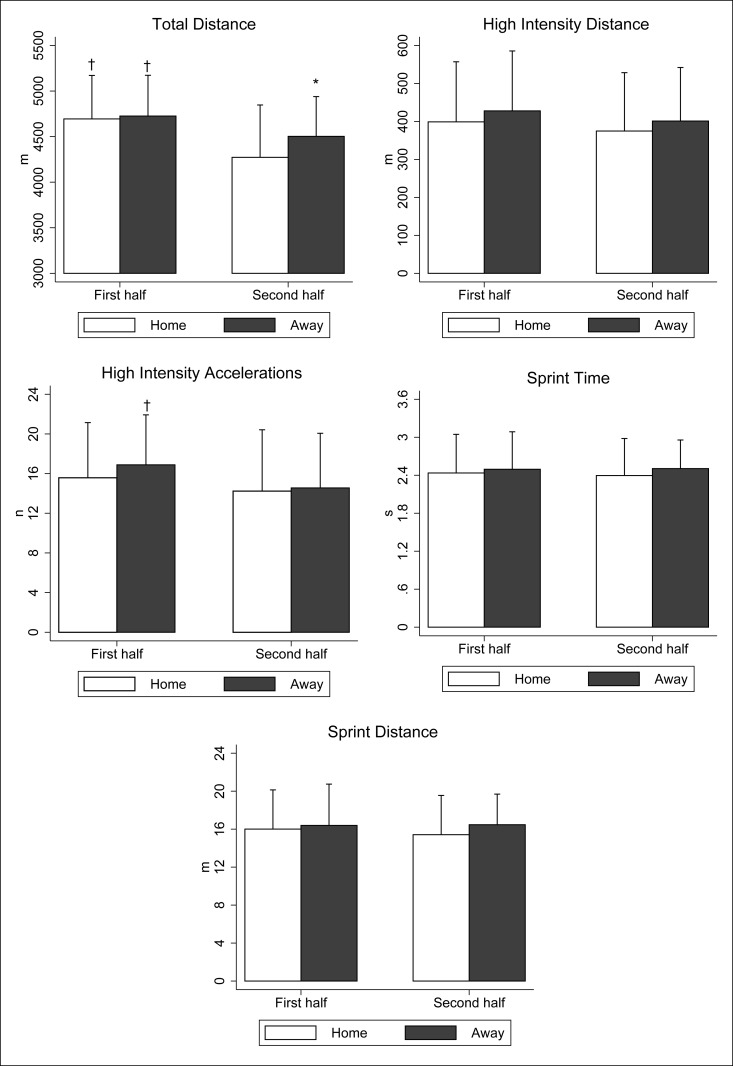
Influence of match location on total distance and high intensity running by 2^nd^ B Division players during the first and second half in football games. The data are presented as mean ± SD. * Significant differences according to the match location (*p*<0.05). ^†^ Significant differences between first and second half (*p*<0.05).

The comparative analysis between matches played at home or away does not reveal significant differences in physical parameters, except in the total distance covered in the second part. The games played away from home showed a greater distance covered by the players in the second half (+ 230.65 m, IC95%: 21.94 to 438.19, ES: 0.46, *p* = 0.031, Fig 1). In relation to the deterioration of performance, differences are only found in the total distance covered and in the high intensity accelerations. The total distance traveled was significantly lower in the second half both in those played at home (-421.44 m, 95% CI: -599.03 to -243.86, ES: 0.80, *p*<0.001) and away (-223.04 m, 95% CI: -411.25 to -34.84; ES: 0.51; *p* = 0.020). On the other hand, the high intensity accelerations were significantly lower in the second half in the games played away (-2.33, 95% CI: -4.49 to -0.17, ES: 0.23, *p* = 0.034).

Based on the quality of the opponent (Table 1), the players did not show significant differences in any performance indicator. However, the comparative analysis between the first and second half revealed a significant reduction in the total distance covered by the players against lower level teams (-290.42 m, 95% CI: -557.82 to -23.01, ES: 0.72, *p* = 0.033) and medium level teams (-374.56 m, 95% CI: -549.21 to -199.70, ES: 0.71, *p*<0.001). The total distance traveled against high level teams does not show differences between the first and second half. In the same way, the number of high intensity accelerations was significantly lower in the second half respect to first half against low level teams (-3.19, 95% CI: -6.25 to -0.14, ES: 0.61, *p* = 0.041).

**Table 1:** Total distance and high intensity running by 2^nd^ B Division players during the first and second half in football games according to the quality of the opposition.

		Low level	Medium level	High level
First Half	Total Distance (m)	4585.37±419.77†	4775.66±471.73†	4674.64±461.35
	High Intensity Distance (m)	407.17±177.98	418.04±154.26	404.63±149.92
	High intensity Accelerations (n)	16.37±5.40†	15.99±5.39	16.59±5.29
	Sprint time (s)	2.51±0.51	2.44±0.66	2.49±0.55
	Sprint Distance (m)	16.47±3.59	15.98±4.57	16.42±4.05
Second Half	Total Distance (m)	4294.95±386.32	4401.21±589.16	4417.22±490.18
	High Intensity Distance (m)	357.23±145.67	398.86±147.93	388.77±152.05
	High intensity Accelerations (n)	13.17±5.10	14.57±6.15	15.25±5.88
	Sprint time (s)	2.36±0.37	2.52±0.6	2.34±0.47
	Sprint Distance (m)	15.39±2.65	16.33±4.19	15.34±3.6

† Significant differences between first and second half (*p*<0.05).

On the other hand, according to the distance regarding the positions needed to maintain the same category (Table 2), the players traveled greater total distances in the first half when there were more points away from the safety position (+235.86 m; IC95%: 49.03 to 422.70; ES: 0.51; *p* = 0.014). The rest of the variables did not show significant differences depending on the distance from the safety position. Finally, the players revealed a significant deterioration in the total distance traveled between the first and second half when being close (-294.84 m, 95% CI: -441.39 to -148.29, ES: 0.60, *p*<0.001) and far (-435.39 m; IC95%: -707.58 to -163.20; ES: 0.90; *p* = 0.002) from the positions of keep category.

**Table 2:** Total distance and high intensity running by 2^nd^ B Division players during the first and second half in football games according to the distance within the points to keep category.

		Far from keep category	Close to keep category
First Half	Total Distance (m)	4889.38±473.49*†	4653.52±445.16†
	High Intensity Distance (m)	443.44±167.87	403.09±154.62
	High Intensity Accelerations (n)	16.83±4.71	16.01±5.52
	Sprint time (s)	2.49±0.54	2.46±0.62
	Sprint Distance (m)	16.34±3.86	16.14±4.35
Second Half	Total Distance (m)	4453.99±495.1	4358.68±534.31
	High Intensity Distance (m)	404.09±156.18	382.16±145.98
	High Intensity Accelerations (n)	14±4.8	14.49±6.14
	Sprint time (s)	2.54±0.5	2.42±0.53
	Sprint Distance (m)	16.51±3.51	15.74±3.82

* Significant differences from lower distances to maintain the category (*p*<0.05).

† Significant differences between first and second half (*p*<0.05).

## Discussion

The main aim of this study was to examine the effects of situational variables and points needed to keep category on physical match performance in professional soccer players. Depending on match location, the results showed that the distance covered during the second half is significantly higher playing away than playing at home. There are a wide range of factors that can be attributed for home advantage, which may include crowd effects, familiarity, travel effects, territoriality, psychological factors, referee bias, rules factors and specific tactics [26]. Additionally, the home advantage is demonstrated when on average there is a higher proportion of games won than lost [16,27], and more goals scored [27]. Therefore, when a team plays away from home it is more likely to be losing, and that would justify having to cover a greater distance in the second half, compared to the games played at home, to obtain a tie or to win the game.

The results show that the total distance covered in professional soccer players is significantly lower in the second half compared to the first half in both match locations (playing at home or away). One reason can be the reduction in muscle glycogen in some muscle fibers observed during a soccer match, which has been suggested to be one of the most important substrates for energy production, and fatigue towards the end of the match [28]. Previous studies confirmed that there is a decline in physical performance in elite soccer players [18,29,30]. Specifically, a significant decrease during the second half in the total high intensity running distance and total sprint distance was observed [29]. In this line, high-intensity running, very high-intensity running and total distance covered in the first half significantly influenced the distances covered in the second half [18]. Moreover, a significant decline between the first and second half was found in both the physical performance and in some technical skills, like involvements with the ball, short passes and successful short passes [30].

The quality of the opposition has been shown to influence physical demands in soccer [11,14,18,31]. It is known that the distance covered by jogging and walking is higher when the quality of the opponent is better [12]. However, in this study these differences were not found in the same way. The results of the present study show that there was a significant reduction in the total distance covered between the first and second half by players when playing against low and medium quality teams. Another result from this study was that the number of high intensity accelerations was lower in the second half against low quality opponents. This is also in accordance with the results of previous research where the amount of high intensity running during matches was higher against high quality opponent teams than against low quality opponent teams [18].

The quality of the opposition and the match location are included in the concept of situational variables, which may influence the teams and players performance during a soccer match [16–18]. In this sense, it is important to highlight what distinguished the style of play in the different European leagues. Thereby, English League is characterized by a direct style of play; Italian league is to be characterized by the defensive tactical rigor; and Spanish league favors the aesthetic side of the game and having greater control over the game. This can be explained mainly to cultural factors. However, strategictactical factors; specific players' skills, and the type of coach are modular factors in the style of play characteristic of each country [32]. Nevertheless, there is a little number of papers in the scientific literature about the topic of physical differences between elite and amateur soccer players. In the French League, no differences were found in the physical performance variables. Differences only was found in small-sided games situations [33].

This study observed that the distance covered by the players was higher when the team was nearer to relegation. However, this effect only occurred in the first half, while in the second half of the match no differences were found. This reason could be that to avoid relegation, the players in the first half try to move more in order to win the match as soon as possible. Teams finishing in the bottom five and middle ten league positions completed more total high intensity running distance compared with teams in the top five league positions [29]. A difference between studies was that the previous one analyzed many teams compared with the one team analyzed in this study. Another reason could be that the top teams are used to winning most of the time, and it has been shown when a team is winning the professional soccer players performed less high intensity activity as compared to when they are losing [12]. More studies are needed to elucidate if the points needed to stay in the category have an influence on the intensity of physical performance performed by soccer players.

There are some limitations in the present study, one limitation is that the sample was composed for soccer players that belonged to the same team, so more studies are needed to confirm the results obtained in this study, and it would also be interesting to complete the results analyzing more players from several teams at the same time. Another limitation can be that the tactical formation during the matches were not taken into account, as it has been shown that the playing formation impacts on very high-intensity running activities with and without ball possession in professional soccer players [34], and it could also affect the physical match performance analyzed in this study.

### Practical applications

The results from the present study offer additional information to coaches, being able to change the style of play of their teams in order to take advantages on the decrease in performance in the second half when playing against teams that are trying to stay up in the category. In addition, the results support new studies related to match performance in the soccer leagues in which professional and semi-professional teams participate, an area with important complexities that has not been practically investigated so far.

### Conclusion

The present study indicates that the physical performance of professional soccer players is influenced by game location, quality of the opposition and points required to maintain the category. The game location impacts on physical performance in a way that playing away matches means that the distance covered in the second half is higher. The total distance covered decreases less between halves when competing against high-level teams. Regarding the points needed to keep category, players covered a higher total distance in the first half when the team position is nearer to relegation than further from relegation.

## Supporting information

S1 File Manuscript dataset. (XLS)
